# Proactive monitoring of changes in the microbial community structure in wastewater treatment bioreactors using phospholipid fatty acid analysis

**DOI:** 10.1016/j.engmic.2024.100177

**Published:** 2024-11-03

**Authors:** Lawson Mensah, Elise Cartmell, Mandy Fletton, Mark Scrimshaw, Pablo Campo

**Affiliations:** a Kwame Nkrumah University of Science and Technology, College of Science, Kumasi, Ghana; b Scottish Water, Castle House, 6 Castle Drive, Dunfermline KY11 8GG, United Kingdom; c UKWIR Limited, 50 Broadway, London SW1H 0RG, United Kingdom; d Department of Life Sciences, Brunel University London, Uxbridge UB8 3PH, United Kingdom; e Cranfield Water Science Institute, SWEE, Cranfield University, Bedford MK43 0AL, United Kingdom

**Keywords:** Phospholipids fatty acid analysis, Microbial community structure, Solids and hydraulic retention times, Temperature, Bioreactor health, Activated sludge

## Abstract

Diverse microbial community structures (MCS) in wastewater treatment plants (WWTPs) are vital for effectively removing nutrients and chemicals from wastewater. However, the regular monitoring of MCS in WWTP bioreactors remains unattractive owing to the skill and cost required for deploying modern microbial molecular techniques in the routine assessment of engineered systems. In contrast, low-resolution methods for assessing broad changes in the MCS, such as phospholipid fatty acid (PLFA) analysis, have been used effectively in soil studies for decades. Despite using PLFA analysis in soil remediation studies to capture the long-term effects of environmental changes on MCS, its application in WWTPs, where the microbial mass is dynamic and operational conditions are more fluid, remains limited. In this study, microbial communities in a controlled pilot plant and 12 full-scale activated sludge plants (ASPs) were surveyed over a two-year period using PLFA analysis. This study revealed that changes in the MCS in wastewater bioreactors could be detected using PLFA analysis. The MCS comprised 59 % Gram-negative and 9 % Gram-positive bacteria, 31 % fungi, and 1 % actinomycetes. The abundances of Gram-negative bacteria and fungi were strongly inversely correlated, with an R^2^ = 0.93, while the fatty acids cy17:0 and 16:1ω7c positively correlated (R^2^ = 0.869). Variations in temperature, solid retention time, and WWTP configuration significantly influenced the MCS in activated sludge reactors. This study showed that WWTP bioreactors can be routinely monitored using PLFA analysis, and changes in the bioreactor profile that may indicate imminent bioreactor failure can be identified.

## Graphical abstract

**Figure:**
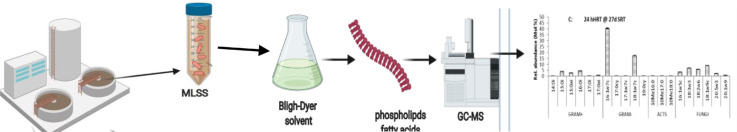

## 1 Introduction

Wastewater treatment plants (WWTPs) have become essential infrastructures in modern society because of their vital role as the last barrier to the removal of contaminants from domestic and industrial wastewater before discharge into surface water bodies. WWTPs employ a wide range of technologies to remove contaminants from wastewater. The underlying principles of these technologies can be categorized into physical, biological, and chemical processes [1]. Physical methods are mainly designed to remove suspended solids, either by sedimentation if they have a sufficiently high settling velocity or filtration if they have a low settling velocity [2]. Chemical processes may involve using a coagulant to neutralize the surface charges on colloidal particles or a precipitating agent to cause an otherwise soluble chemical species to become insoluble and enhance its removal via physical methods. However, biological processes rely on the accumulation of microorganisms in a bioreactor to degrade chemicals and nutrients in wastewater, including trickling filters, activated sludge, and rotating biological contactors. Most biological WWTPs in developed countries are activated sludge plants (ASPs). Their performance is highly influenced by the microbial community structure (MCS) in the aerated lanes where the activated sludge (also known as mixed liquor) resides [3]. The MCS of an activated sludge reactor depends on several parameters. According to a recent study [4], the influent concentrations of ammonia, total phosphates, and chemical oxygen demand are the most influential parameters in determining the composition of ASP microbes in South Africa. The solid and hydraulic retention times (SRT and HRT, respectively) have also been shown to affect the microbial composition of ASP bioreactors [5]. The MCS is also significantly influenced by temperature [6,7], WWTP configuration [8], dissolved oxygen (DO) concentration [9], and altitude [7]. However, despite the critical role of bioreactors in the treatment of wastewater, their content and microbial communities are not regularly monitored.

Technological advancements in instrumentation and molecular techniques have allowed researchers to study the effects of wastewater processing parameters and conditions on the MCS in engineered systems [10]. This has led to a corpus of knowledge regarding the MCS of different bioreactors in a high-performance state and during treatment failure [11,12]. Having baseline knowledge of the microbial composition of WWTP reactors and how they change with time and process variations will allow wastewater treatment scientists to detect significant variations that may lead to bioreactor failure and avert them or manipulate bioreactors to achieve optimum performance.

Failures of WWTPs to meet regulatory standards are very common [13]. In 2020, WWTPs serving 12 agglomerations of towns and cities in the United Kingdom with a population of approximately 100,000 were found to be noncompliant with regulatory standards for their effluent discharge [14]. The UK Environment Agency is currently investigating 2200 WWTPs for noncompliant effluents in accordance with their discharge permits [15]. These failures to comply, resulting in the discharge of untreated or partially treated wastewater to receiving water bodies such as streams, rivers, and seas, have serious environmental and human health consequences [16]. The European Union reported that >50 % of European water bodies have poor ecological status as a direct result of receiving >6.5 % of their total flow from WWTP effluent [17]. Despite the high cost of these treatment failures to the environment, wastewater corporations, and human health, investment in monitoring bioreactor health remains the least preferred option in comparison to monitoring effluent quality and failsafe systems. Although high-resolution DNA sequencing methods such as Polymerase Chain Reaction Denaturing Gradient Gel Electrophoresis (PCR-DGGE) in environmental samples are expensive and require a high level of skill, they can generate enormous amounts of data for bioinformatics analysis and interpretation [18]. Despite the great insights provided by utilizing these high-resolution MCS assessment methods in WWTPs, limitations prohibit wastewater companies from routinely monitoring the MCS in their treatment reactors to understand the health of the bioreactor. It is therefore necessary for alternative low-cost, less technical, but fast and effective molecular methods such as phospholipid fatty acid (PLFA) analysis to be developed or adopted for assessing the MCS in WWTP bioreactors. Moreover, there have been several instances where changes in MCS were unidentifiable through PCR-based molecular methods or community-level physiological profiling but were detected via PLFA analysis [19].

Microbial cellular membranes contain PLFAs, which are produced in response to changes in environmental conditions and stress [20]. PLFAs maintain the integrity of cell membranes; therefore, microorganisms in similar conditions produce matching PLFAs in their membrane structures, irrespective of their geospatial location. Genes for phospholipid synthesis were conserved because they are essential for survival [21]. When a microbial cell dies, the breakdown of phospholipids in the cell membrane is rapid, which makes the characterization of the fatty acid profile in the soil an effective tool for determining the viable microbial mass [22].

Qualitative and quantitative assessments of PLFAs in microbial cell membranes have been used as biomarkers for MCS in soil and activated sludge for decades [[23], [24], [25]]. However, these two environmental samples have different microbial compositions and abundances [26,27]. Microbial groups such as Gram-positive (Gram+) bacteria, Gram-negative (Gram-) bacteria, actinomycetes, and fungi have been studied to identify the predominant fatty acids that make up their cellular membranes and the changes that occur in their fatty acid profiles during environmental stress [21]. The fatty acids used in the estimation of Gram+ bacterial abundance are 14:0i, 15:0i, 15:0ai, 16:0i, 17:0i and 17:0ai; cy17:0, cy19:0, 15:1ω4, 16:1ω7c, 16:1ω9c, 17:1ω9c, 18:1ω7t and 18:1ω9c for Gram- bacteria; 10Me16:0, 10Me17:0, and 10Me18:0 for actinomycetes; and 16:1ω5c, 18:1ω9c, 18:2ω6, 18:3ω3, and 20:5ω3 for fungi [21,22]. These fatty acid notations were written according to the fatty acid nomenclature described previously [28].

Changes in the microbial environment in wastewater treatment systems, such as seasonal temperature variations or alterations in influent quality arising from factory closures or openings, affect the type and abundance of phospholipids synthesized in the microbial cell membrane and cell wall [29]. In ASPs, changes in the parameters and operational conditions, such as the type and abundance of nutrients, SRT, HRT, temperature, and dissolved oxygen levels, lead to changes in the phospholipid profile of the microbial cells. Several studies [25,30,31] have used PLFA analysis to assess changes in MCS due to variations in temperature, SRT, HRT, and specific oxygen uptake rate. Although the use of PLFA analysis to assess microbial community composition in the soil is still relevant and effective, research on its use in wastewater treatment systems is lagging and scarce. PLFA analysis has additional advantages compared to DNA sequencing techniques, such as being cheaper, less technical, and generating less data, making it a deployable tool for the routine monitoring of MCS in WWTP bioreactors. In this study, we monitored changes in the MCS of 12 municipal ASPs and one pilot-scale activated sludge plant using PLFA analysis to characterize the effects of DO concentration, temperature, SRT, and HRT on the MCS.

## 2 Materials and methods

### 2.1 Full-scale activated sludge plant configuration and operation

Twelve full-scale municipal ASPs with population equivalents ranging from 25,000 to 1.75 million in England were selected (Supplementary Table S1). Each full-scale plant operates several primary sedimentation tanks in parallel, followed by activated sludge (aerated) lanes and final sedimentation tanks. Two ASPs had a tertiary treatment sand filtration system attached, whereas the other had a moving bed bioreactor for pre-treatment prior to the aerated lanes. Table 1 summarizes the operational parameters and configurations of the pilot- and full-scale ASPs, and detailed information about the full-scale plants and operational conditions during each sampling visit is provided in Table S1.

**Table 1:** Population equivalent, plant layout descriptions and operational conditions of full-scale activated plants studied.

WWTP	PE	Configuration of the full-scale activated sludge plants	DO (mg/L)	Temp ( °C)	HRT (h)	SRT (d)
**A**	**105,000**	Primary sedimentation tank, nitrifying ASP and final sedimentation tank	1.74 ± 0.6	16.0 ± 3.2	10.8 ± 2.0	9.80 ± 1.5
**B**	**71,100**	Primary sedimentation tank, nitrifying ASP and final sedimentation tank	1.46 ± 0.5	15.9 ± 3.4	13.5 ± 3.1	11.5 ± 4.9
**C**	**147,975**	Primary settling tank, MBBR, nitrifying ASP and sand filtration	0.94 ± 0.8	17.9 ± 2.2	11.4 ± 3.1	9.64 ± 3.0
**D**	**25,000**	Primary sedimentation tank, nitrifying ASP, final sedimentation tank and lagoons	1.27 ± 0.5	16.5 ± 1.4	19.3 ± 4.4	11.7 ± 4.8
**E**	**55,761**	Primary sedimentation tank, anoxic / oxic ASP and final sedimentation tank	1.27 ± 0.7	14.0 ± 2.9	19.7 ± 6.8	9.10 ± 3.8
**F**	**409,918**	Primary sedimentation tank, nitrifying ASP and final sedimentation tank	1.47 ± 0.6	16.4 ± 2.5	10.5 ± 2.5	14.0 ± 3.4
**G**	**302,000**	Primary sedimentation tank, anoxic / oxic ASP and final sedimentation tank	3.34 ± 2.6	16.2 ± 2.5	12.6 ± 3.3	13.8 ± 3.7
**H**	**97,029**	Primary sedimentation tank, nitrifying ASP and final sedimentation tank	2.60 ± 2.3	15.7 ± 2.9	9.10 ± 2.3	10.8 ± 2.6
**J**	**122,127**	Primary sedimentation tank, anoxic / oxic ASP, final sedimentation tank and sand filtration	2.00 ± 1.8	14.1 ± 3.0	14.9 ± 3.1	8.10 ± 1.9
**K**	**101,000**	Primary sedimentation tank, nitrifying ASP and final sedimentation tank	1.65 ± 0.1	13.3 ± 2.3	17.6 ± 1.0	17.6 ± 3.9
**L**	**66,205**	Primary sedimentation tank, biological nutrient removal plus phosphorus removal, nitrifying ASP and final sedimentation tank	2.60 ± 2.0	15.8 ± 3.0	10.4 ± 4.3	9.30 ± 2.8
**M**	**1,750,000**	Primary sedimentation tank, nitrifying ASP and final sedimentation tank	1.10 ± 0.2	16.7 ± 2.9	10.2 ± 2.2	11.0 ± 0.7

P.E. = Population equivalent; MBBR = moving bed bioreactor; DO concentration was measured in the aeration tank.

### 2.2 Pilot plant setup and operation

A pilot-scale activated sludge plant comprising a 180-L primary settling tank, 360-L aerated tank, and a 100-L final settling tank received raw sewage pumped from the inlet chamber of a nearby trickling filter treatment plant (Table 2). The initial inlet flow rate was set to 45 L/h to achieve a constant HRT of 8 h in the aerated basin. The SRT was varied to 3, 10, and 27 d by controlling the amount of return-activated sludge (RAS) wasted while recycling 60 % of the suspended solids exiting the aerated tank. The RAS, MLSS, and effluent SS were assessed daily to determine the SRT. For the HRT experiments, the SRT was maintained at 27 d, while the flow rate was increased to 22.5 L/h and later to 15 L/h to achieve 16 and 24 h HRT, respectively (Table 2). The stabilization period for each setup was equivalent to a minimum of one SRT, during which consistent sanitary performance was observed in the reactor.

**Table 2:** Configuration and operational condition of the pilot plant activated sludge plant.

Configuration and operational conditions of the pilot-scale ASP	Experimental variables	**SRT (d)**	**HRT (h)**
	Low SRT; short HRT	3	8
	Medium SRT; short HRT	10	8
	Long SRT; short HRT	27	8
	Long SRT; medium HRT	27	16
	Long SRT; long HRT	27	24

PST = primary settling tank; FST = final settling tank.

### 2.3 Sample size and collection

In the pilot-scale study, 35 samples were collected in triplicate from the aerated base of the pilot plant. After the stabilization period, 1 L of the activated sludge in the aerated basin was sampled in clean plastic containers for seven consecutive days. Samples were immediately preserved in an ice chest at 4 °C and transported to the laboratory for analysis. In this study, 62 samples were collected from 12 full-scale ASPs over an 18-month period. Then, 2.5-L MLSS samples were taken from the activated sludge lanes, transferred into plastic bottles, stored in cool boxes at 4 °C, and sent to the laboratory for analysis. At least two samples from each ASP were collected per season (winter and summer) using a dip can on a metal pole following the grab sampling method.

### 2.4 Physicochemical analysis of mixed liquor samples

During sample collection, the temperature and DO levels of the mixed liquor in the bioreactor were measured in situ using a Hanna HI-991,401 pH meter (Digitalmeters, UK) and Hach HQ40d dissolved oxygen meter, respectively. The operational conditions of the WWTPs were also recorded to enable the calculation of the SRT and HRT. Total suspended solids in all mixed liquor samples were determined by following Standard Methods for the Examination of Water and Wastewater [32].

### 2.5 Phospholipid fatty acid analysis

#### 2.5.1 Chemicals

The Bligh-and-Dyer solvent was prepared by mixing 210 mL citrate buffer, 260 mL chloroform, 530 mL methanol, and 30 mg butylhydroxytoluene. Analytical-grade ammonium acetate, ammonium hydroxide, chloroform, methanol, and toluene (Sigma Aldrich, UK) were used as solvents. Citrate buffer was made by dissolving 28.8 g of anhydrous citric acid and 44 g of tri-sodium citrate in a liter of ultrapure deionized water. The other reagents prepared included a 1:1 methanol-toluene mixture, 50 mL methanol containing 0.01 mol potassium hydroxide, 59 mL/L acetic acid in H_2_O, and a 4:1 hexane-chloroform mixture.

#### 2.5.2 Sample preparation and analysis

The MLSS (250 mL) was transferred to a Nalgene bottle in the laboratory. The sample was centrifuged for 20 min at 8000 rpm at 4 °C, and approximately 90 % of the supernatant was decanted. The centrate was re-suspended in the remaining supernatant, poured into a 50-mL plastic sample bag, and stored at −80 °C for 48 h Prior to PLFA analysis, the sample was freeze-dried at −50 °C. Phospholipids (0.5 g) from the freeze-dried MLSS were leached with Bligh-and-Dyer solvent and separated using solid-phase extraction. The phospholipids were then reacted with methanol under alkaline conditions to convert them into fatty acid methyl esters. Finally, the phospholipids were quantified using gas chromatography with a flame ionization detector, as previously described [33], with modifications [20].

### 2.6 Statistical treatment of data

The recorded temperature, DO levels, SRT, and HRT of the bioreactor at the time of sampling were compared using MANOVA in Statistica 13 software. Changes in the MCS, as depicted through variations in the PLFA profile, were assessed using principal component analysis and the post hoc Tukey HSD.

## 3 Results and discussion

### 3.1 Microbial community structure determination in the pilot-scale setup at various SRT and HRT

**Fig. 1:**
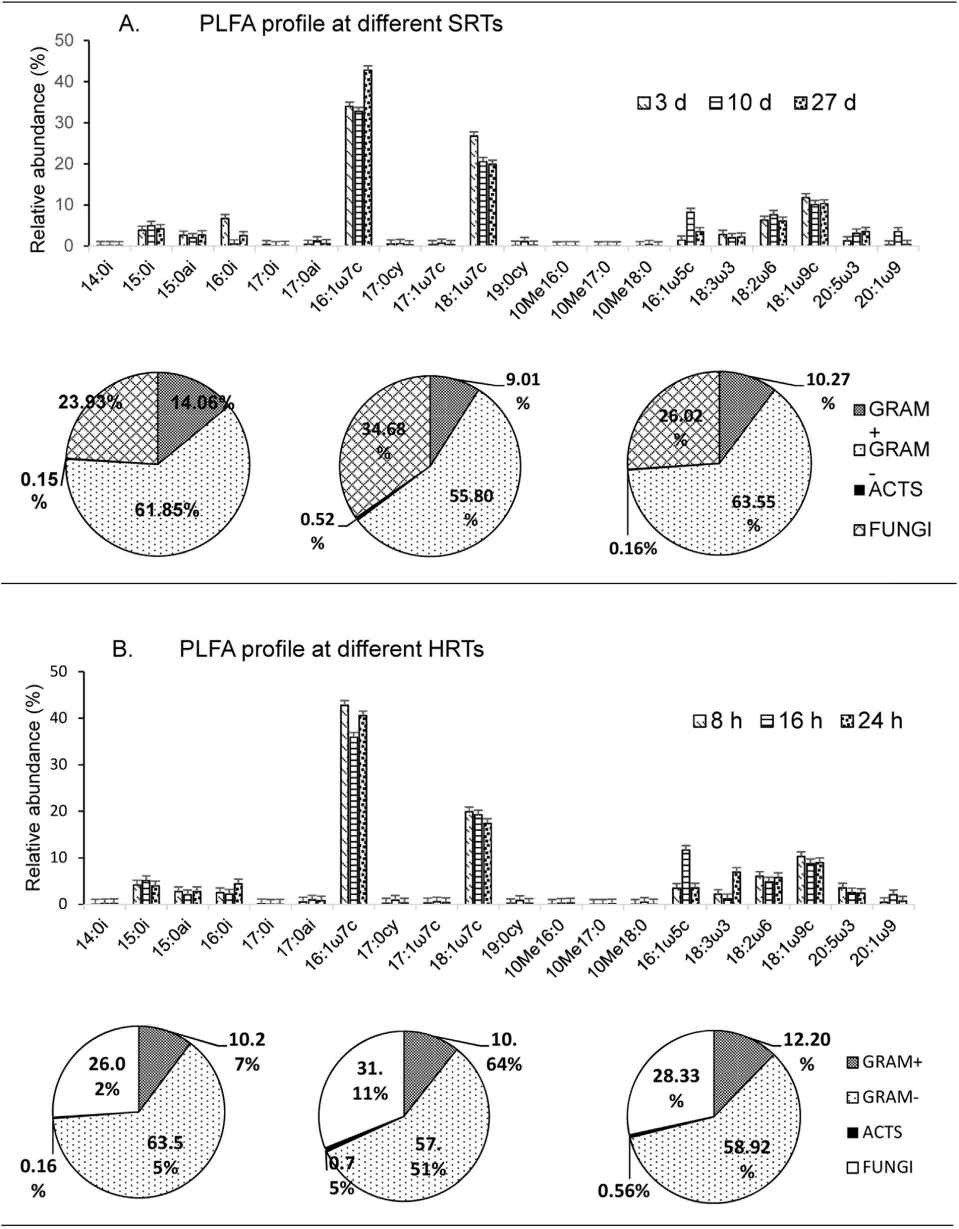
Phospholipid fatty acid levels at [A] 3, 10, and 27 d SRT and [B] 8, 16, and 24 h HRT.

Gram-negative bacteria were dominant in all setups, followed by fungi, Gram-positive bacteria, and actinomycetes. The highest mean abundances of fatty acid methyl esters formed from the phospholipids in the microbial cell membranes were 16:1ω7c (37.2 %; range: 23.7 to 42.8 %), 18:1ω7c (20.8 %; range: 17.4 to 20.8), and 18:1ω9c (9.99 %; range: 8.76 to 11.78 %) as in Fig. 1. The fatty acid 16:1ω7c is predominantly found in Gram- bacteria such as *Pseudomonas* and *Vibrio*, switching between the synthesis of the *cis* and *trans* isomers in response to varying temperatures [34]. An increase in the SRT affects the MCS (Fig. 1), which may lead to a variation in the α-diversity in pilot-scale bioreactors [5]. The variation in the fatty acid profiles with increasing SRT and HRT was significant (*n* = 5, *p* = 0.003). An increase in the SRT from 3 to 10 d increased 16:1ω5c fatty acid levels 5.5-fold, decreased 16:0i levels by 92 %, and resulted in Gram+ bacteria decreased by 5 % and Gram- bacteria abundance decreased by 6 %. Moreover, the fungal population increased by 10.7 %, and the abundance of actinomycetes increased from 0.15 % to 0.52 % (*p* = 0.0014, *n* = 7). A statistically non-significant variation in Gram+ bacterial abundance was observed when the SRT was further prolonged to 27 d (*p* = 0.059, *n* = 7), while significantly reducing the abundance of actinomycetes (*p* = 0.0052, *n* = 7) and fungi and increasing the abundance of the Gram- bacterial population by 7.8 %. These observations may be explained by starvation or stress in the bioreactor as the SRT increases, inducing microorganisms to modify their phospholipid fatty acids in their membranes as a response, such as increasing the proportion of cy17:0 and 16:1ω7c fatty acids in their cell membrane [35].

Fig. 1 (panel B) also shows that an increase in the HRT affects the MCS. Changing the HRT from 8 to 16 h increased the fungal population by 5 % and decreased the abundance of Gram- bacteria by 6 %. A longer HRT (24 h) insignificantly increased Gram-negative bacteria (1.4 %) and reduced the abundance of fungi and Gram+ bacteria by 2.8 % and 1.6 %, respectively.

### 3.2 Microbial community structure in full-scale ASPs

**Fig. 2:**
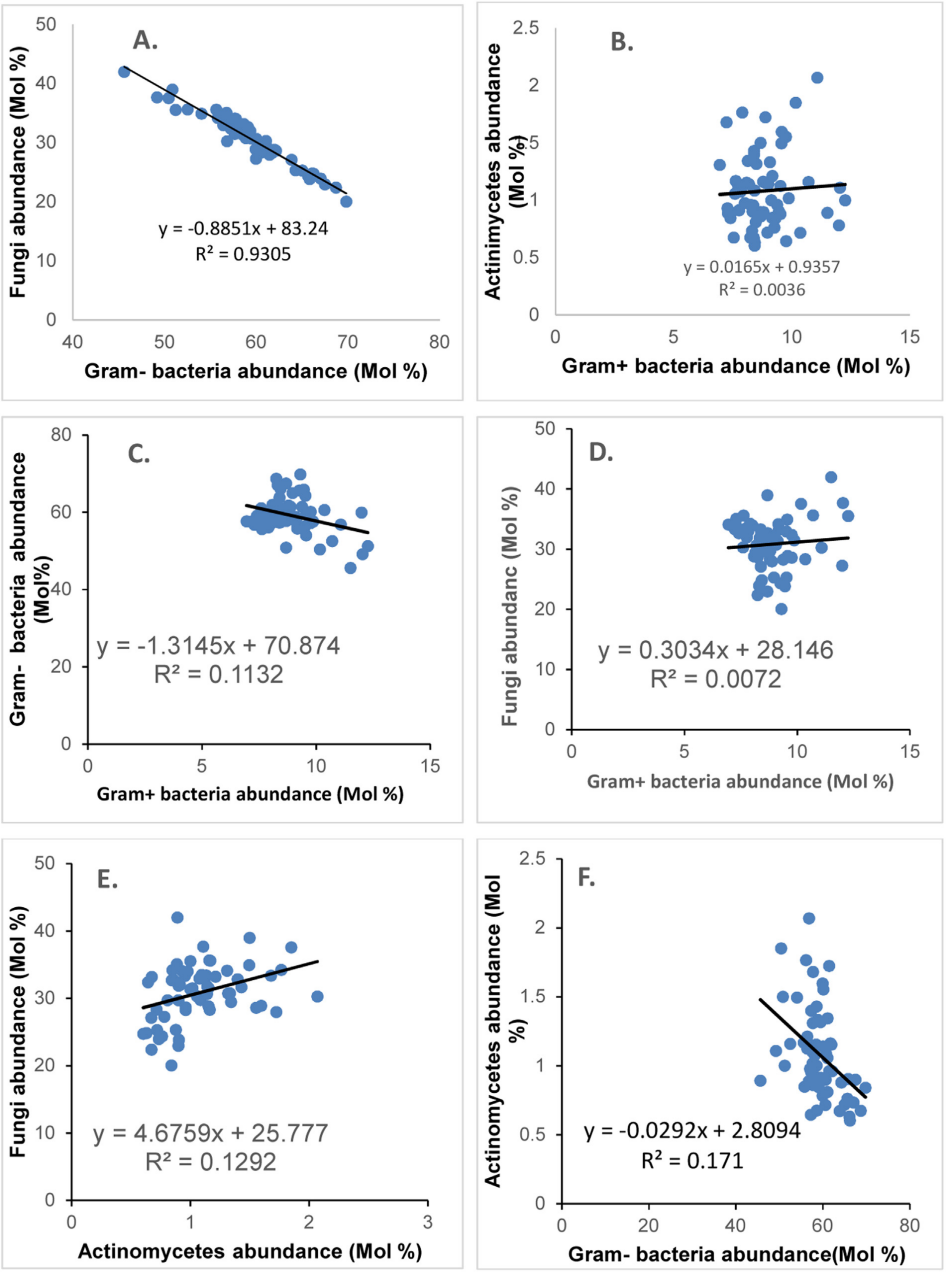
Linear regression analysis between the abundance of the microbial groups assessed in the full-scale activated sludge plants: [A] Gram- bacteria against fungi, [B] Gram+ bacteria against actinomycetes, [C] Gram+ bacteria against Gram- bacteria, [D] Gram+ bacteria against fungi, [E] actinomycetes against fungi, and [E] Gram- bacteria against actinomycetes.

**Fig. 3:**
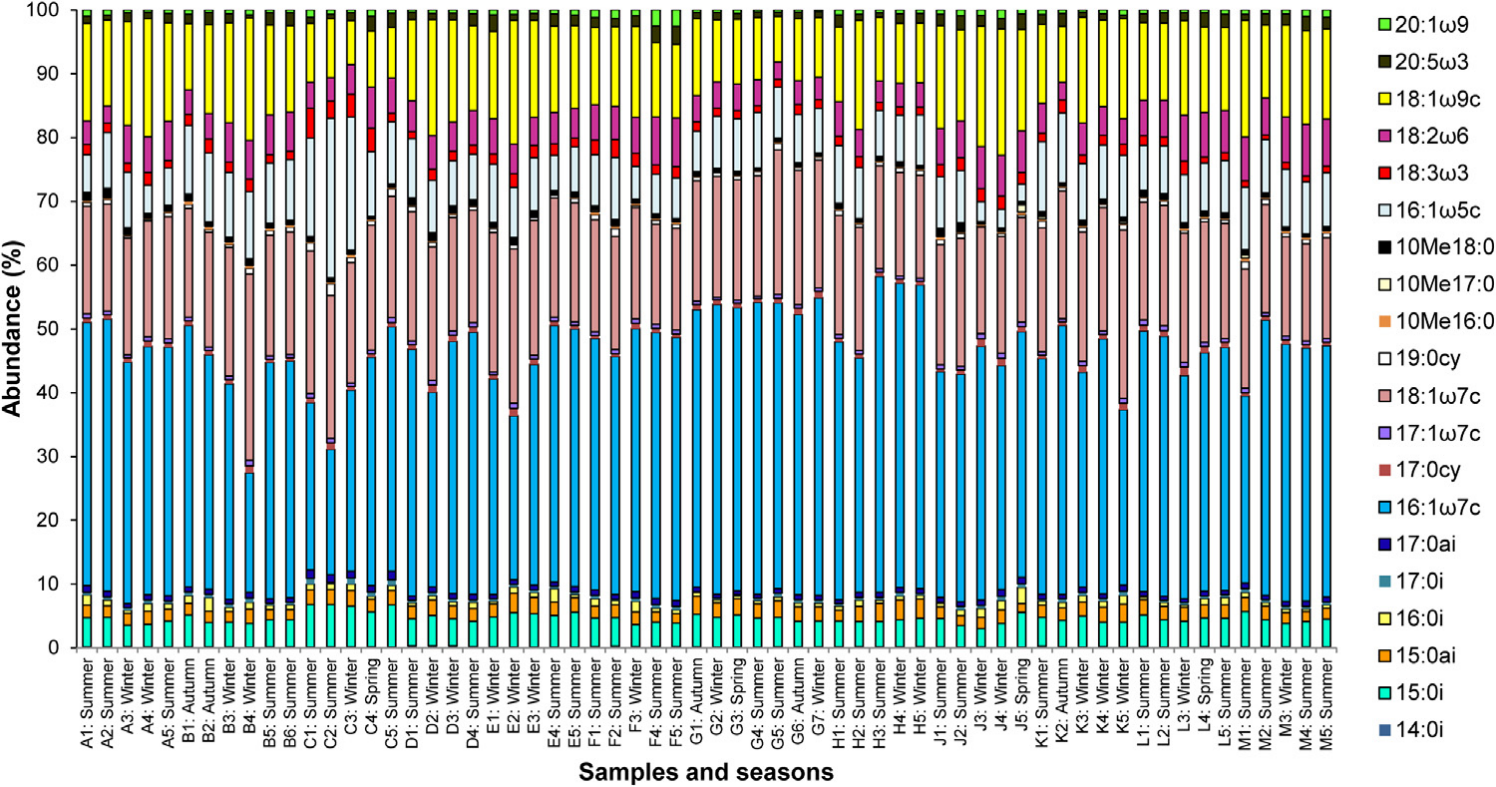
Variation in fatty acids methyl ester abundances of mixed liquor sample from full-scale activated sludge plants as biomarkers of microbial community structure in the treatment reactor. Footnote: A1: Summer represents first sample from WWTP A which was taken in the summer season.

The MCS followed a similar trend in abundance as the pilot-scale ASP, with Gram+ bacteria as the dominant group and actinomycetes as the least abundant. Linear correlation analysis between the abundances of the microbial groups showed that Gram- bacteria and fungi were strongly negatively correlated (R^2^=0.93; Fig. 2, panel A). The most abundant fatty acids in the microbial cell membranes were in the order 16:1ω7c > 18:1ω7c > 18:1ω9c > 16:1ω5c > 18:2ω6 > 15:0i; 14:0i, 10Me16:0, and 10Me17:0 (Fig. 3). Gunina et al. [29] also found that 16:1ω7c and 16:0 were the most abundant fatty acids in four full-scale ASPs. In this research, the highest 16:1ω7c level was found in sample H3, comprising approximately 50 % of the total fatty acids extracted. However, samples B4:Winter (fourth sample from WWTP B taken during winter) and C2:Summer (second sample from WWTP C taken during summer) had 18:1ω7c and 16:1ω5c as the most abundant fatty acids, respectively, with 16:1ω7c being the least abundant, at approximately 19 % each. Sample C2 (summer) also showed a significantly higher abundance of fatty acids 19:0cy compared to all other samples. All samples from WWTP C uniquely contained high levels of the fatty acid 18:3ω3, which has been linked to an abundance of fungi in biofilms [34]. The abundance of Gram- bacteria ranged from approximately 46 to 70 %, consistent with other studies [29,36,37]. The abundance of fungi, the second most dominant microbial group, ranged from 20 to 42 %, followed by Gram+ bacteria, with 7.0–12.3 % abundance. Actinomycetes were the least abundant microbial group, with an abundance ranging from 0.6 to 2 %. The abundances of Gram- bacteria in WWTPs A and B were 58–62 % and 51–58 %, respectively. WWTP A contained a relatively low abundance of the fatty acid 16:1ω5c (6 %). The MCS in the bioreactors of plants C and D contained Gram- bacteria at abundances between 46 % and 60 % and 54 % and 61 %, respectively. WWTP C contained the lowest levels of Gram- bacteria and the highest fungal abundance of 27.2–42 %. This result was attributed to the unique layout of the plant, which incorporates a moving bed bioreactor (MBBR) in the plant layout prior to the activated sludge lanes. MBBRs are highly effective for BOD and COD removal from settled sewage, leading to low organic loading and subsequent starvation and stress in the bioreactor [38]. These conditions are similar to those produced at high SRT in the pilot-scale study. Hence, it was noted with interest that the three fatty acids with high abundance at 27 d SRT (16:1ω7c, 10Me16:0, and 20:5 ω3) were also highly abundant in some samples from WWTP C. The biomass of WWTP E comprised 52–61 % Gram- bacteria, 28–36 % fungi, 8–40 % Gram+ bacteria, and 0.7–1 % actinomycetes. The bioreactor of WWTP F contained 0.8–1.4 % actinomycetes, 7–9 % Gram+ bacteria, 30–33 % fungi, and 57–61 % Gram- bacteria. On average, samples from WWTP G had the highest abundance of Gram- bacteria (64–70 %) and the lowest abundance of fungi (20–25 %). Mixed liquor samples from WWTP H contained 58–66 % Gram- bacteria and 23–33 % fungi, whereas samples from WWTP J had a narrow range of Gram- bacterial abundance (56 %–59 %), with WWTP K containing between 56 and 64 % abundance. In sample K5 from this plant, the abundance of Gram- bacteria was 56 %, consistent with the other samples from WWTP K. However, the levels of 16:1ω7c decreased significantly from approximately 40 to 27 %, whereas the levels of 18:1ω7c increased from its typical abundance of 20 % in WWTP K to approximately 26 % (*p* < 0.05). The abundances of Gram- bacteria in the samples from WWTPs L and M were 58–62 % and 50–62 %, respectively. Fungal abundance at site L was 28–33 %, whereas that at site M was 29–38 %. The order of abundance according to microbial group was similar to that in other environmental samples [39].

### 3.3 Influence of temperature, SRT, HRT, DO concentration, and plant configuration on the MCS in full-scale ASPs

**Fig. 4:**
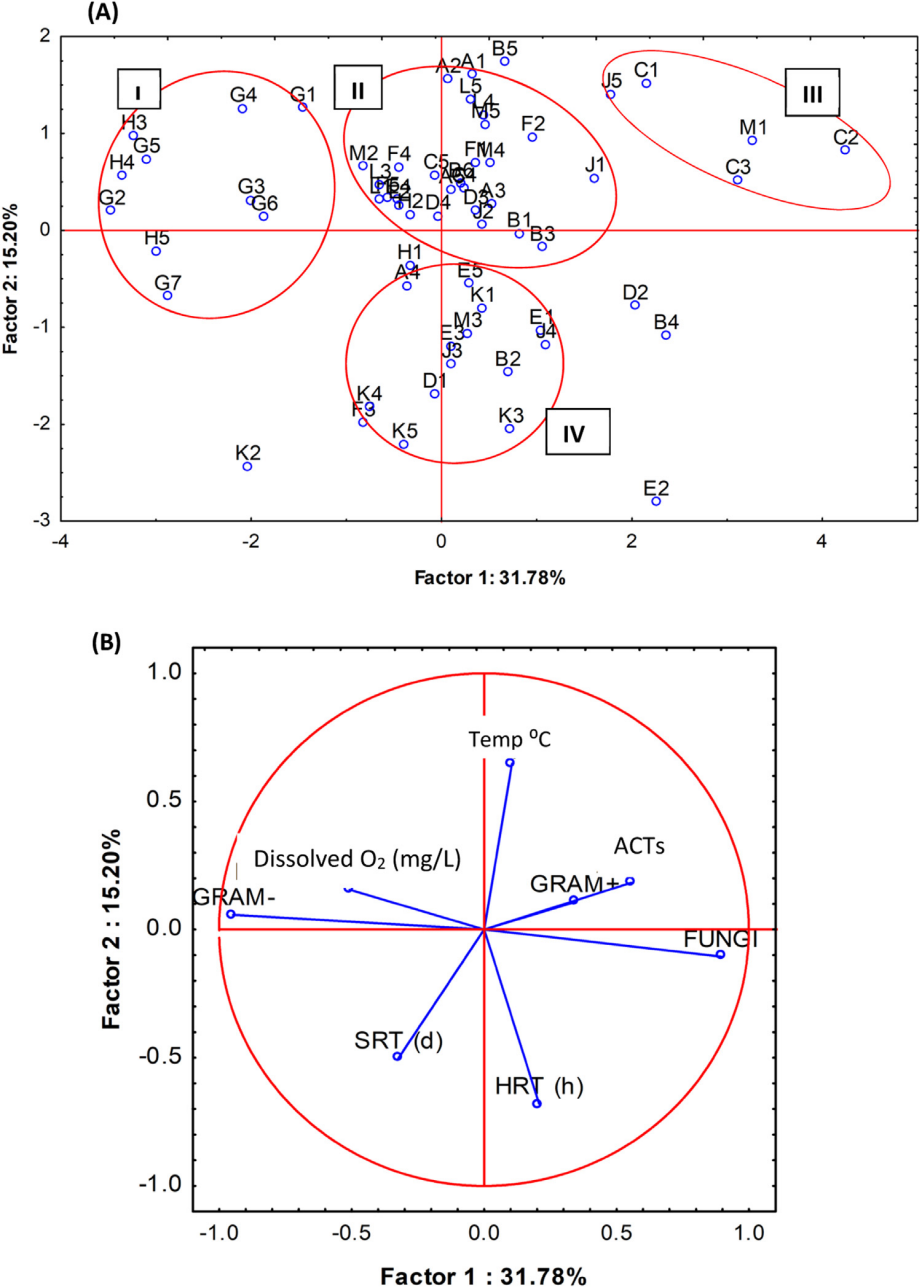
[A] Clustering of full-scale ASP samples according to their microbial community structure. [B] Influence of SRT, HRT, DO, and temperature on the microbial community structure.

Changes in temperature, SRT, HRT, and DO concentration in ASPs had a significant impact on the microbial cellular fatty acid profiles and MCS, in agreement with the findings of Xu et al. [40]. The MCS in the 12 full-scale ASP samples were clustered into four groups based on their fatty acid profiles (Fig. 4, panel A), and as influenced by temperature, SRT, HRT, and DO levels (Fig. 4, panel B). Group **I** consisted of samples that contained a high abundance of Gram- bacteria, low fungal abundance, and occurred under high DO concentrations. Groups **II** and **IV** contained most of the samples and were from bioreactors operating at low to mid ranges of SRT (6.5 to 12.5 d) and HRT (8 to 10 h). The main difference between them may be that Group **II** samples were taken at warmer temperatures (> 14 °C) while Group **IV** corresponded to colder temperatures (< 14 °C). Group **III** comprised samples with relatively high abundances of Gram+ bacteria, actinomycetes, and fungi, which were collected at warmer temperatures and low DO concentrations, e.g., samples C1, C2, C3, J5, and M1. The samples from WWTP C and G clustered in opposing groups owing to their contrasting abundances of fungi and Gram-positive and Gram-negative bacteria. Samples from WWTPs G and H, collected at relatively low temperatures and with high DO levels, had high numbers of Gram-negative bacteria and low fungal abundances.

Samples from WWTPs C, G, and M were clustered into separate groups to show their differences in fatty acid profiles from other WWTPs. Temperature strongly influenced the fatty acid profile. However, the SRT was the most influential process parameter that caused shifts in the fatty acid profiles and MCS. This is because the SRT directly determines the food-to-microorganism ratio, and a high SRT can lead to starvation and stress in WWTP reactors [41]. Increased SRT significantly affected the abundance of Gram+ bacteria and actinomycetes, whereas the abundance of fungi was negatively affected by high DO concentrations. Plant configuration was also found to affect the MCS in full-scale activated sludge reactors. Thus, of the 12 WWTPs studied, the configuration of WWTP C was unique because it included an MBBR before the nitrifying activated sludge lanes (Table 1). This unique configuration corresponded to a different MCS in its samples, as their mean abundance of fatty acid 16:1ω5c was the highest and that of 16:1ω7c was the lowest, in contrast to all the other WWTPs (Fig. 3). Consequently, most samples from WWTP C were found in Group **III** (Fig. 4, panel A).

This study has shown for the first time that PLFA analysis can be used to simultaneously assess the influence of process parameters such as SRT, HRT, DO concentration, and seasonal temperature changes on the fatty acid profile and MCS in full-scale ASPs in a way that may be applicable to the wastewater industry. Relying on the analysis of effluent quality (as is currently done) to determine whether a failure has occurred or is prominent is often too late. Instead, by proactively monitoring the MCS aside from the daily SRT and flow rate records, significant changes will be detected early so that their causes can be identified. Thus, temperature, HRT, SRT, influent quality, or DO concentration changes may also be identified. The early detection of significant MCS changes that may lead to WWTP failure can be averted with appropriate interventions, such as increasing or decreasing the SRT, HRT, and DO levels, or dosing with a chemical reagent. Proactive monitoring of the MCS will minimize the occurrence of WWTP failure, as it does in the information technology and petrochemical sectors, where minor issues with the potential to develop into major challenges are detected early and resolved through software updates and maintenance [42,43]. The benefits of proactive monitoring in WWTPs include no discharge of untreated wastewater, compliance with discharge permits, no fines or regulatory penalties, and maintaining a good corporate reputation [42]. The cost of monitoring the MCS using PLFA is low and can be maintained in-house, as the level of expertise and equipment required is not excessive. For the routine monitoring of bioreactor health (MCS) in activated sludge plant reactors, regular sampling of mixed liquor followed by PLFA analysis should be incorporated into the weekly assessment of WWTPs.

## 4 Conclusion

The MCS comprised approximately 59 % Gram- bacteria, 9 % Gram+ bacteria, 1 % Actinomycetes, and 31 % fungi in the pilot- and full-scale activated sludge bioreactors operated under different conditions investigated in this study. The predominant phospholipid fatty acids identified from the biomass of the activated sludge were 16:1ω7c, 18:1ω7c, and 18:1ω9c. Under carefully controlled SRTs and HRTs, PLFA analysis detected differences in the MCS in the pilot-scale ASPs, and the observed variations were influenced by changes in the SRT and HRT. The fatty acids cy17:0 and 16:1ω7c were strongly and positively correlated (R^2^ = 0.869) as the SRT increased from 3 to 27 d WWTP configuration, SRT, and seasonal temperature changes were the most influential parameters in determining the MCS in bioreactors in full-scale ASPs; these were also detectable using the low-cost PLFA method. The fatty acids 16:1ω7c, 18:1ω7c, 18:1ω9c, 16:1ω5c, 18:2ω6, and 15:0i were the most abundant in 97 % of the activated sludge samples. Moreover, the abundances of Gram- bacteria and fungi were negatively correlated (*R* = 0.95). This study showed that PLFA analysis is versatile in tracking changes in the MCS in WWTP bioreactors under experimental conditions and in full-scale settings, indicating that PLFA analysis can be used for routine bioreactor health assessment in WWTP reactors.

## Data Availability Statement

The data used to support the findings of this study have been included in the article.

## CRediT authorship contribution statement

**Lawson Mensah:** Writing – original draft, Investigation, Data curation. **Elise Cartmell:** Funding acquisition, Conceptualization. **Mandy Fletton:** Project administration. **Mark Scrimshaw:** Supervision. **Pablo Campo:** Supervision.

## Declaration of Competing Interest

The authors declare that they have no known competing financial interests or personal relationships that could have appeared to influence the work reported in this paper.

## Appendix Supplementary Materials

Image, application 1
